# Latent human traits in the language of social media: An open-vocabulary approach

**DOI:** 10.1371/journal.pone.0201703

**Published:** 2018-11-28

**Authors:** Vivek Kulkarni, Margaret L. Kern, David Stillwell, Michal Kosinski, Sandra Matz, Lyle Ungar, Steven Skiena, H. Andrew Schwartz

**Affiliations:** 1 Department of Computer Science, Stony Brook University, Stony Brook, New York, United States of America; 2 Melbourne Graduate School of Education, The University of Melbourne, Melbourne, Australia; 3 Judge Business School, University of Cambridge, Cambridge, United Kingdom; 4 Graduate School of Business, Stanford University, Stanford, United States of America; 5 Columbia Business School, Columbia University, New York, United States of America; 6 Computer and Information Science, University of Pennsylvania, Philadelphia, Pennsylvania, United States of America; University of Vermont, UNITED STATES

## Abstract

Over the past century, personality theory and research has successfully identified core sets of characteristics that consistently describe and explain fundamental differences in the way people think, feel and behave. Such characteristics were derived through theory, dictionary analyses, and survey research using explicit self-reports. The availability of social media data spanning millions of users now makes it possible to automatically derive characteristics from behavioral data—language use—at large scale. Taking advantage of linguistic information available through Facebook, we study the process of inferring a new set of potential human traits based on *unprompted* language use. We subject these new traits to a comprehensive set of evaluations and compare them with a popular five factor model of personality. We find that our language-based trait construct is often more *generalizable* in that it often predicts non-questionnaire-based outcomes better than questionnaire-based traits (e.g. entities someone likes, income and intelligence quotient), while the factors remain nearly as *stable* as traditional factors. Our approach suggests a value in new constructs of personality derived from *everyday human language use*.

## Introduction

*What are the fundamental characteristics that make a person uniquely him or herself?* Psychology has long tried to answer this question by deriving the latent factors that distinguish people, are relatively stable across time and populations, and predict meaningful outcomes [1, 2, 3, 4]. While several different models of human personality exist, the most dominant model is the Big 5 or Five Factor Model, in which personality characteristics generally group into five factors: extraversion, agreeableness, conscientiousness, neuroticism/ emotional stability, and openness/ intellect [5, 6]. The Big 5 is meant to summarize, at a broad level, characteristic behaviors that distinguish a person throughout different contexts of their daily life. It is typically assessed through a questionnaire, in which a person reflects on their typical thoughts and behaviors [5]. Such questionnaires only indirectly capture actual behavior and also suffer from systematic response biases [7, 8].

The rise of big data offers opportunities to study everyday behavior at a scale never before possible. Each day, people reveal aspects of their lives through words expressed online through social media, such as Facebook and Twitter. Leveraging this linguistic information, we derive a trait model based on everyday linguistic behavior. Our approach analyzes the words and phrases of tens of thousands of users and their millions of messages to infer a set of traits.

In line with trait theory [9], we seek a small number of generalizable and stable traits that capture meaningful differences between people. We call these *behavior-based linguistic traits (BLTs)*. Our method does not rely on any hand-crafted lexica or questionnaires and it scales well to leverage the large amount of data available on social media. While some have leveraged social media and open-vocabulary techniques to assess *existing* trait models—e.g., Big 5 [5] of bloggers [10] or Facebook users [11], the dark triad [12] using Facebook [13]—to the best of our knowledge, none have attempted to *infer* the latent traits themselves.

We evaluate BLTs along two criteria:

- **Generalizability**: The factors need to be generalizable across a large variety of predictive tasks.
- **Stability**: The factors should be relatively stable over time and populations. The factor scores of users over time should be correlated, at levels similar to cross-time and cross-population correlations of the Big 5.

The overall goal of this study is to determine if it is feasible to derive *generalizable* and *stable* traits from the behavior of social media language use. We produce *BLTs* using methods of matrix factorization. Then, we evaluate the extent to which the *BLTs* meet this criteria by considering their predictive validity, test-retest validity, dropout reliability, face validity, and ability to predict psychological variables (Depression Scores) and social-demographic variables (IQ, Income and Likes).

## Background

Personality theory and research has a long history of finding characteristics, factors, and aspects that distinguish people from each other. Although personality has been used in a myriad of ways, much of the research can generally be classified into two purposes: (a) Identifying the major constructs and factors that consistently distinguish groups of individuals, and (b) Predictive models that either predict personality traits from other characteristics or use personality to predict other variables. We further discuss each of these themes below.

### Modeling personality

Personality psychologists have long sought to identify fundamental characteristics that ideographically distinguish individuals yet also adequately cluster across groups of individuals to reveal consistent patterns of behavior. While there are multiple approaches for studying individual differences, the most dominant approaches are rooted in the lexical hypothesis, which suggests that key features of human personality will become a part of the language that we use to describe ourselves [1, 4, 14]. The importance of language in psychology is underscored by [15]:

Language is the most common and reliable way for people to translate their internal thoughts and emotions into a form that others can understand. Words and language, then, are the very stuff of psychology and communication.

Consequently, a long line of work in personality psychology has sought to characterize individual differences based on words that people use. The Big 5 model arose by having people rate themselves across dictionary-based adjectives (e.g., I see myself as extraverted, enthusiastic; reserved, quiet), and then using factor analysis to group responses into a small number of factors. Early on, a lexicon of 18, 000 words that distinguish one person from another based on an English dictionary was proposed by [1]. Participants rated themselves on these characteristics, and numerous factor analyses revealed anywhere between three and 16 major factors. Other lexica and lists of adjective descriptors have also been developed and tested over the past century. Although there are some exceptions, across multiple questionnaires, cultures, and temporal periods, the Big 5 factors consistently appear [6].

As such, the dominant approach towards characterizing personality is based on hand-crafted lexica and dictionaries [4]. Psychologists have developed a variety of questionnaires that capture these personality traits [4, 5, 16]. People can easily reflect on their own personality, or others can provide an evaluation of that person’s personality using these questionnaires.

The five factors are hierarchical in nature, with the five factors underscored by aspects which are comprised of specific traits or facets, which reflect habitual behaviors, thoughts, emotions, and ways of responding to situations [17]. Personality questionnaires are meant to summarize everyday behavior, but represent a reflection based on self (or other) perceptions, rather than directly assessing everyday behavior as it occurs. Underlying characteristics better predict outcomes [18], but also are less consistent across individuals. As the Big 5 do successfully predict important life outcomes, they are useful in providing broad representations of relatively stable individual differences, at the expense of capturing behaviors that occur in everyday life.

Other approaches to personality assessment exist. For instance, narrative approaches to personality (e.g., [19]) successfully provide rich idiographic descriptions of a person within their everyday context. However, such approaches are time and resource intensive, and thus only occur at small scale, making it a challenge to find common nomothetic constructs.

An initial attempt was made to use an open-ended approach to learn personality traits by extracting common themes from self-narrative texts [20]. Using a dataset of 1, 165 open-ended self-descriptive narratives, a factor analysis was performed on the most frequently used adjectives to reveal latent factors. Latent factors were shown to correlate moderately with the Big 5 factors and suggested psychologically meaningful dimensions. The current study extends this work at much larger scale.

### Predictive models of personality

Personality is interesting in part because it is predictive of meaningful life outcomes, including education and job success, social relationships, physical and mental health, and longevity (e.g., [21, 22]). Personality can also be predicted by other biological, social, psychological, and behavioral measures.

With recent advances in computational social science, researchers have sought to predict personality, as well as other psychosocial and demographic differences, amongst users [10, 23, 24, 25, 26, 27, 28, 29, 30, 31]. In this paper, we focus on work related to the description and prediction of personality from language on social media.

Multiple studies have analyzed social media data, including Facebook posts, text messages, and Twitter tweets, finding correlates of the Big 5 and other individual characteristics with language [24, 25, 32]. Park et al. developed a language-based personality assessment based on Facebook language that predicted personality at similar levels to other methods of personality assessment [29]. Golbeck et al. proposed a method to predict personality of a user based on their posts on Twitter [26]. Sumner et al. proposed a method to predict dark personality traits, based on Twitter language [27]. Plank and Hovy analyzed 1.2 million Tweets and proposed a model to predict Myers-Briggs personality types [28].

While successful, studies increasingly suggest various complexities. Iacobelli et al. analyzed bloggers and found that the best performing model combined multiple linguistic features, including stemmed bigrams and common words [10]. Notably, they also highlighted the need for more refined and complex linguistic features. Recent works show that moving beyond words and incorporating complex features like distributed sentence representations improve personality prediction [30, 31]. As a whole, studies suggest that language can predict personality, but simplistic models might fail to adequately capture the complexities of the human psyche.

While existing studies begin with personality models as the ground truth, the current study harnesses the power of social media data to examine traits that arise from the language itself.

## Materials and methods

In this section, we describe the details of our dataset, our proposed method to learn latent factors and the design of experiments to evaluate the derived factors.

### Ethics statement

All participants explicitly acknowledged consent for their responses and Facebook information to be used for research purposes. All research procedures were approved by the University of Pennsylvania Institutional Review Board.

### Datasets

We used a dataset of 20,356,117 Facebook status messages over 152,845 distinct users obtained using a custom application [33]. We filtered out all users who posted less than 1000 words overall, are over 65 years of age, or who claimed that they are not from the US. Additionally, we filtered out all messages that are not English.

Among these users, 49,139 have data on age, gender and their Big 5 personality scores (Big5), based on 20 personality items from the International Personality Item Pool (IPIP) [34] which comprises our final data set. A few sample messages are shown below:

- goodbye to anybody i didn’t get to catch up with before i go away.
- a calm mind makes u see things in a better shade whose existence was merely ignored
- scars heal, greatness fades and all that one is left behind are the memories made

About 62.8% of the users in our dataset are female. The age distribution is skewed towards younger people with the median age of 22 years and a mean age of 25.49 years. While we believe that our derived latent factors should capture various ages and genders, we also investigate residualizing out demographic factors (age and gender). All messages were tokenized and stop-words (very frequent words like “the” or “is”) were removed in the pre-processing stage.

### Factor generation

We considered several methods to learn our latent factors. Before describing our proposed method, we discuss a few alternative methods that we considered to learn the latent factors.

#### Alternative methods

- **Latent Dirichlet Allocation (LDA)**: We first modeled the compiled linguistic information from each user as a document and latent factors as topics. LDA [35] enables us to learn these factors (i.e., topics) that represent each person based on the corpus of the user’s text. Each user is then represented as a mixture over the learned factors. We used the MALLET [36] toolkit to learn LDA factors, set *α* = 5, and enabled hyper-parameter optimization. We optimize the hyper-parameters every 20 iterations with a burn-in of 10 iterations. LDA represents each user as a probability distribution over topics (factors). Since factor scores are always non-negative and bounded by summing to 1, the scores per person have the undesirable quality that they are always negatively correlated with each other (e.g. a single person could never be high or low in all factors, a constraint that does not occur in typical factor analyses or reflect personality theory). This motivated us to explore approaches without such a bound for this first exploration of modeling a small number of traits from social media.
- **Singular Value Decomposition (SVD)**: We next considered SVD [37]. We specified a user-set (U) and a finite set of vocabulary terms corresponding to those users (V). We constructed a term-user matrix *M* and computed a low-rank approximation of *M* using SVD.
  While SVD does not have the drawbacks of LDA, we observed that a more general version of dimensionality reduction, “factor analysis (FA)”, demonstrates better empirical performance on predictive tasks and motivates FA as our proposed method to capture traits. As such, our proposed methodology draws on FA, rather than LDA or SVD.

#### Proposed method: Factor analysis (FA)

Factor Analysis (FA) has long been a part of psychological research and the development of psychometric assessments. The method seeks to represent a set of variables as linear combinations of a small number of latent factors. Formally, given a matrix *M*, factor analysis seeks to learn latent factors *F* and a loading matrix *L* such that:

$$\begin{array}{c} M=FL+E \end{array}$$ (1)

where *E* represents an error matrix. While SVD seeks to learn factors that account for all of the variance, FA is more general and learns factors that account for the common variance but allows for some unexplained residual variance. In fact, SVD can be interpreted as a special case of Factor Analysis (FA) under the conditions of isotropic noise [38].

Typically in psychology, factors are estimated and then rotated to find the best fitting solution. We chose to use FA as an approach to identify latent user factors. We applied FA on the User-Term matrix and investigated various rotations of the loading matrix L to obtain potentially more interpretable factors. In our final models, we used a promax (oblique) rotation with equa-max rotation for all our experiments.

**Fig 1:**
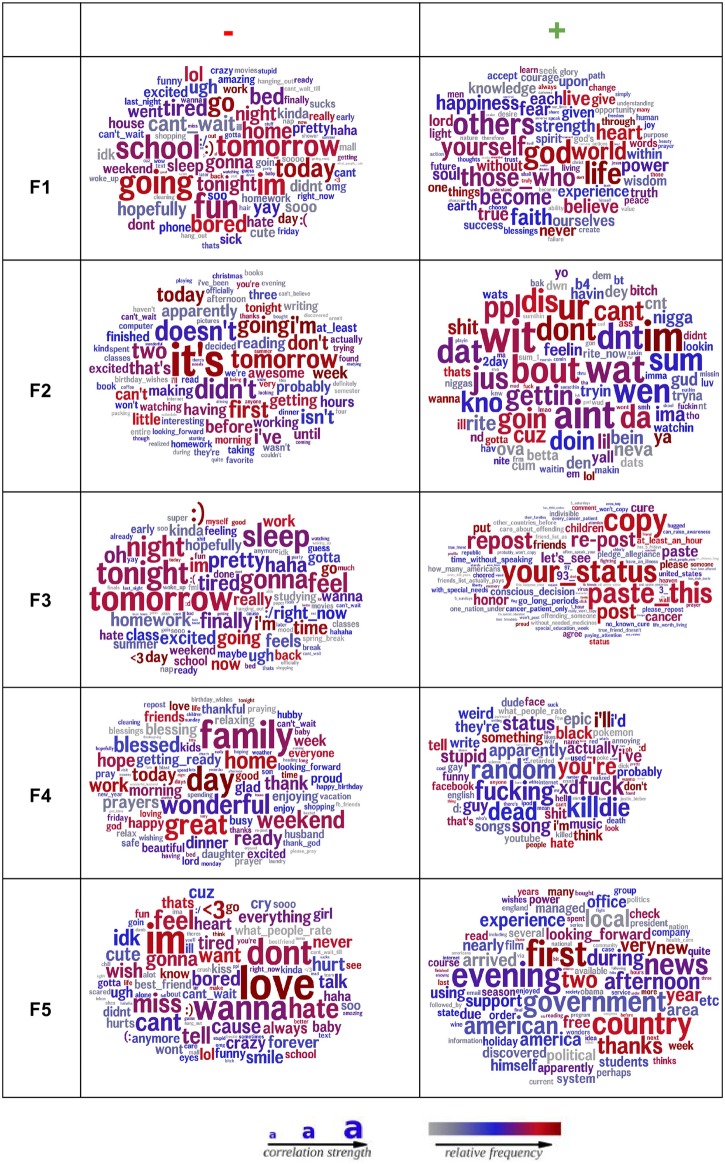
Word clouds showing the most/least correlated words for each factor (with rotation). Word clouds showing the most/least correlated words for each FA factor (with rotation) as obtained using Differential Language Analysis ([11]). The larger the word, the more strongly it correlates with the factor (For all word clouds shown, FDR correction has been done to only show significant words. Also spatial location does not code for anything.). Color indicates frequency (grey = low use, blue = moderate use, red = frequent use).

Using factor analysis, we extracted 5 factors. While we noted that a Scree test using acceleration suggested 3 factors, we consider 5 factors, so that we can make a fair comparison against Big5. We chose to extract 5 factors as a direct comparison to the Big5 model. For comparison, we also extracted 10 and 30 factors (aligning with 10 underlying aspects [17] and 30 facets [5]; results of these models are available in the Supplemental Information section. Fig 1 illustrates word clouds corresponding to the most and least correlated words for each factor (i.e., different language analysis; [11]). The factors capture emotion words such as life, heart, happiness, love (see FA:F1(+)) and non-emotion words such as week-end, school, work, tomorrow, tonight (see FA:F1(-)). These observations suggest that our factors capture a variety of behavioral and psychological cues.

## Differential analysis and convergent validity

**Fig 2:**
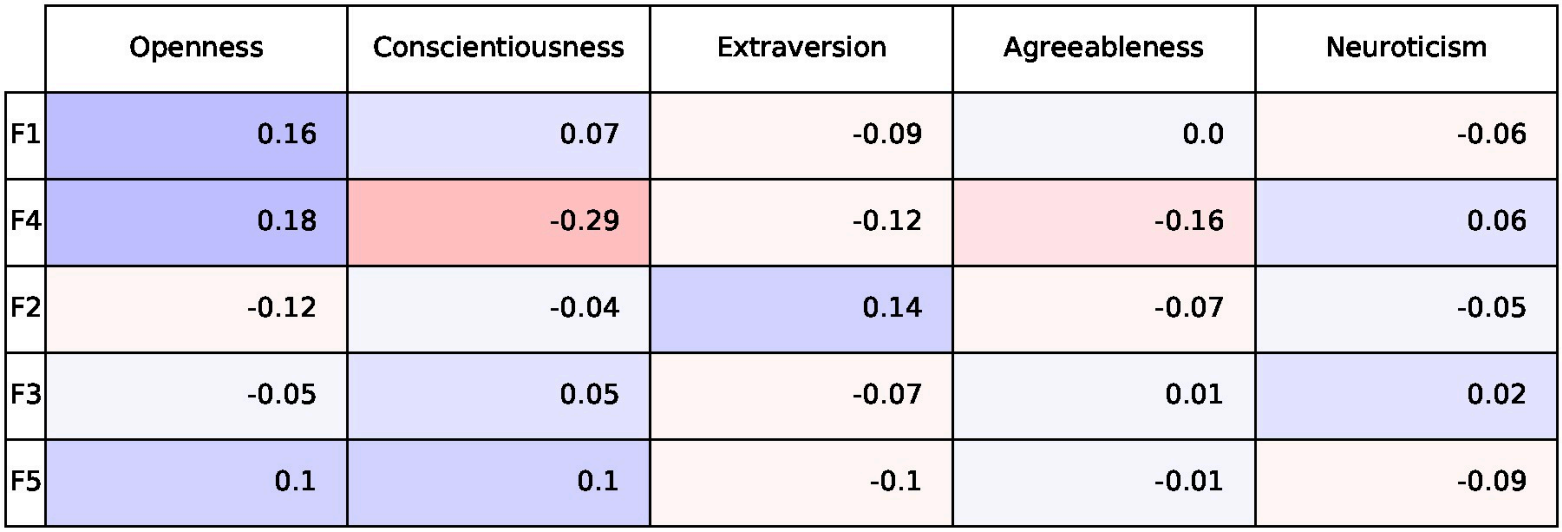
Correlations between the learned factors and the Big5 factors.

We next explore how our derived factors relate with one another and with the Big5, using Pearson correlation coefficients (r). If the factors are capturing real personality characteristics, they should be correlated to some extent with the personality factors that have dominated personality research. But if they capture unique variance, then correlations should only be low to moderate. They should also minimally correlate with one another. As illustrated in Fig 2, the factors most strongly correlate with extraversion and openness. F4 demonstrates the strongest correlation, reflecting lower levels of conscientiousness. The F4 words are similar to [32], with numerous swear words on the positive side and family, work, and relaxation words on the negative side [32].

**Fig 3:**
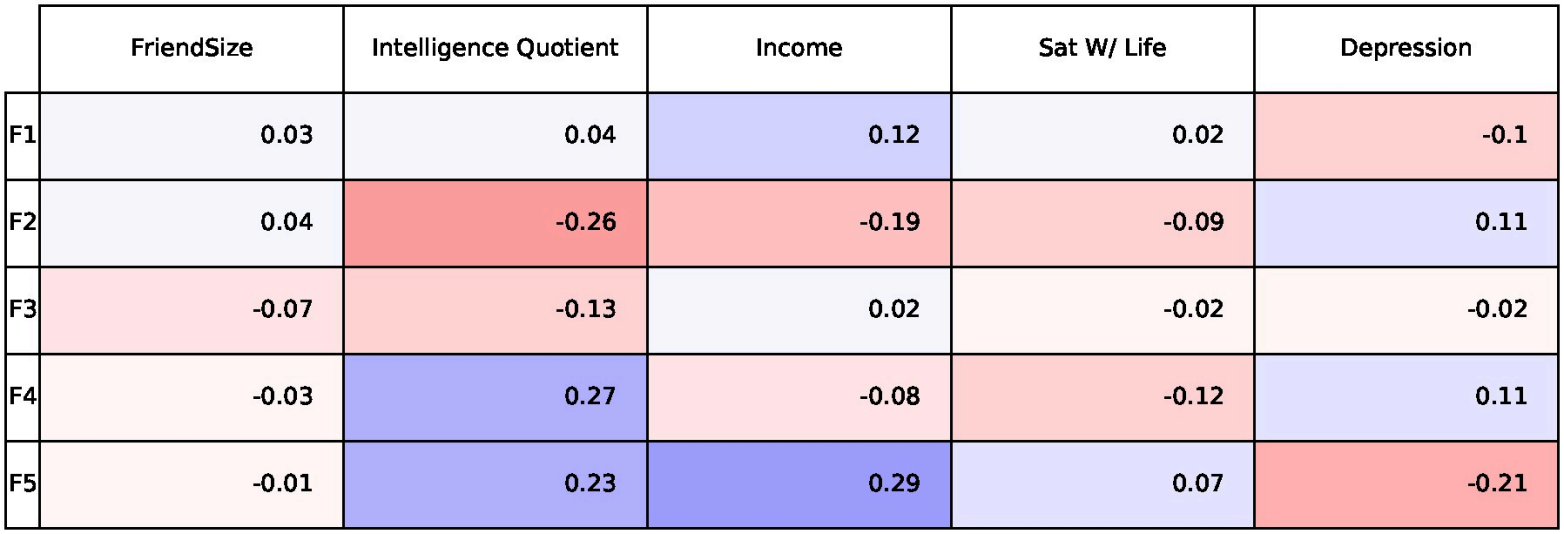
Individual factor correlations with outcomes. Note how F4 which captures the use of swear words negatively correlates with Satisfaction with Life (SWL).

We also report the correlation of each individual factor with outcomes which we show in Fig 3. In general, we note that each individual factor correlates slightly with outcomes but we also observe stronger correlations. Observe for example, that Factor F2 which appears to capture the behavior of teenagers correlates negatively with income. Furthermore, observe how Factor F4, which captures people who are offended (and use swear words) is negatively correlated with Satisfaction with Life (SWL).

**Fig 4:**
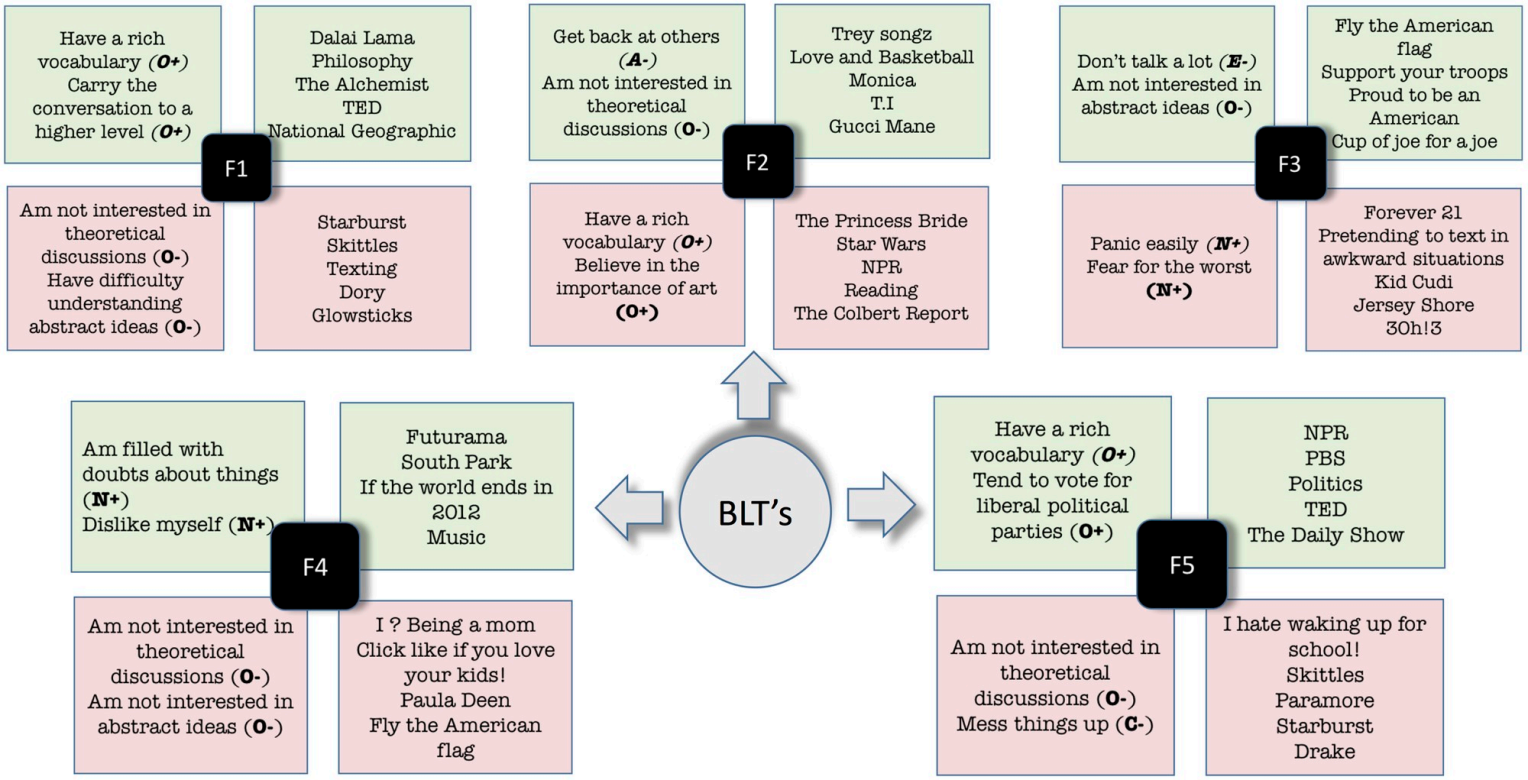
Questions (left of each factor) and Likes (right of each factor) that correlate the highest (green) and lowest (pink) for each of our 5 behavioral-linguistic trait factors.

We now show the 2 Big5 Questions and the 5 Likes that correlate the most positively and negatively with each individual factor in Fig 4 which reveal some insights into the human behaviors captured by our factors. To give a couple of examples, Factor F1 captures traits of people who have a rich vocabulary and like to engage in deeper conversations. Note that people with these traits also like philosophy and like Dalai Lama, The Alchemist and TED. On the other hand, people with a low factor F1 score are not interested in theoretical discussions, have difficulty understanding abstract ideas, and like animated movies such as Finding Dory. Similarly note that factor F5 also captures openness and a liberal outlook, where people who have a rich vocabulary tend to vote for liberal political parties and watch/listen to NPR, PBS and The Daily Show.

In summary, all of these examples suggest that our learned factors capture a variety of human behavior as observed on social media, entirely derived from their language using an open vocabulary approach.

**Fig 5:**
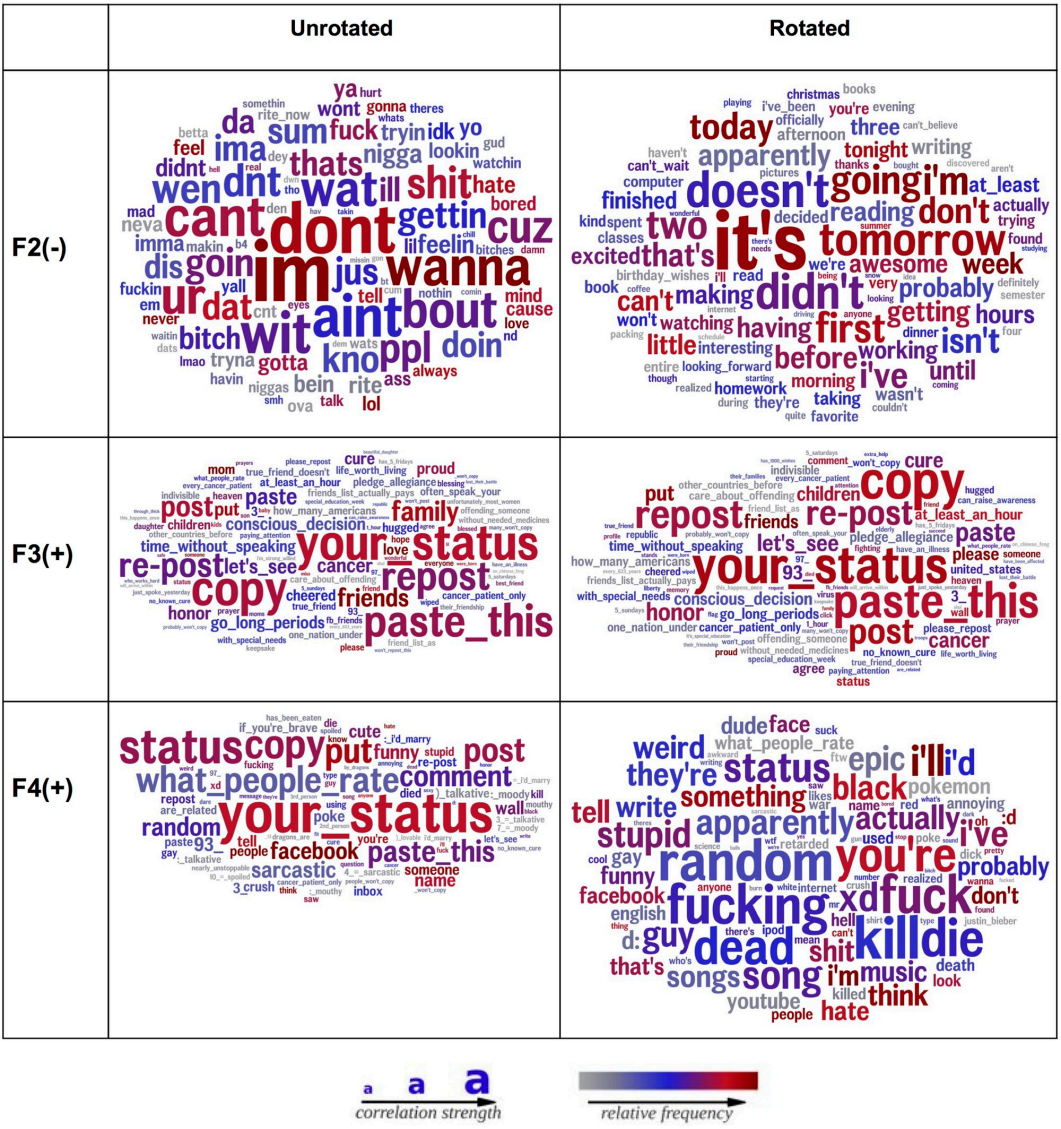
Word clouds showing the effect of a rotation. Word clouds showing the effect of a rotation. A rotation yields markedly distinct factors. Note the absence of words like “paste this” in the rotated version in multiple factors as opposed to the unrotated version where multiple factors are characterized by words like “paste this” and “status update”. The larger the word, the more strongly it correlates with the factor. Color indicates frequency (grey = low use, blue = moderate use, red = frequent use) [11].

Finally, Fig 5 illustrates the effect of rotation on the factor structure. While the un-rotated version has multiple factors that are characterized by words like “paste this” and “status update”, note the stark absence of words like “paste this” in the rotated version in multiple factors thus yielding a more distinct factor structure.

## Evaluation

In this section, we present methods to comprehensively evaluate our derived factors. Our evaluations broadly seek to quantify two aspects of the factors: *generalizability* and *stability*.

### Generalizability

Predictive validity seeks to measure the *generalizability* of the learned factors by measuring their predictive performance on a number of tasks, based on other data available on the same users. We group these evaluations into two categories: *questionnaires/survey* and *behavioral/ economic* outcomes. For each outcome, we predict the outcome using regression, and report our performance using the Pearson r correlation coefficient (except in the case of categorical classification where we report AUC).

#### Behavioral/Economic outcomes

1. FriendSize: The number of friends was pulled from users’ Facebook profiles.
2. Income: Estimated income was available through a 5 minute long questionnaire administered to 2, 623 Facebook users in the US which includes questions on age, gender, educational qualifications and income. Due to the skewed distribution of income, we use the logarithm of reported income.
3. Intelligence Quotient (IQ): Users completed an IQ test through a custom platform [33]
4. Likes: We predict a small number of broad categories that a users like, such as Rock Music Bands, Gaming, and Hobbies. To determine these categories, we began with a matrix *N* of Users and fine-grained categories of likes on Facebook. We considered only the top 10, 000 likes by popularity (i.e., frequency counts across users). We then clustered the users with Non-negative matrix factorization (NMF) to reduce the dimensionality of *N* to 20 clusters (which we evaluated qualitatively). As an illustration, we show one such broad level cluster which corresponds to music bands in the metal genre of music: Disturbed • System of a Down • Linkin Park • Slipknot • Avenged Sevenfold • Breaking Benjamin • Bullet for my Valentine • Metallica • Korn

#### Questionnaire/Survey outcomes

1. Life Satisfaction (SWL or Sat. W/ Life): Satisfaction with life (SWL) was obtained through the 5-item Satisfaction with Life questionnaire [39].
2. Depression Ratings: Depression was obtained through the 20-item Center for Epidemiological Studies-Depression (CES-D) questionnaire [40].
3. Additional Big 5 Questions: Beyond the 20 personality items used to calculate the big5 scores, many users had between 10 and 80 additional personality items available. The evaluative task is to predict personality based on these additional items. We consider only Questions 21–100, since the first 20 questions were directly used to compute their Big5 scores. This, thus examines predictive validity over and above basic Big5 correlations.

#### Learning predictive models

For regression tasks we use Linear Regression with *L*2 penalty (Ridge Regression), and for classification tasks we train a Logistic Regression classifier. In both cases, we restrict analyses to linear models to ensure that our models are interpretable and reveal the inherent predictive power of the factors. We set our hyper parameters using a grid search and use cross-validation to provide more stable estimates and to quantify model variance. We report our results as the mean performance over 10 different random splits of training and test data.

### Stability

#### Test/Retest validity

We evaluate the stability of the learned factors by conducting a test-retest experiment. Our experimental procedure is as follows:

1. Split the entire corpus of messages into two parts: (a) a training portion used for learning a model to infer factors (75% of the corpus) and (b) a test portion (25% of the corpus) which is held out from training to test the estimated model. We further divide each user’s posts into several time periods (6 months apart). To ensure enough data, we only consider users who have written at-least 1000 words within the time-frame. Past work [29, 41] has empirically established this number of words as sufficient for language based assessments of personality.
2. Learn a model to infer factors for each user using the training set.
3. Infer factors for users in each time period of the test set.

We report the correlations across available time periods. Park et al. [29] found that language-based assessments of the Big5 factors demonstrated strong correlations over time, ranging from r = .62 for neuroticism to r = .74 for openness across consecutive 6 month intervals, with lower correlations across farther time periods [29]. If the BLT factors demonstrate endurance, then we’d expect to see strong correlations on the test set (r = .60 and above) across a 6 month interval, with some declines across subsequent temporal periods.

#### Dropout reliability

Finally, we evaluate the external validity of the learned factors across different samples of users. The learned factors should not be overly dependent on a specific set of users from the training data. We quantify the sensitivity of the learned factors to the presence (or absence) of users as follows:

1. We randomly drop 20% users from the training data before we learn the factors.
2. We repeat step 1 one hundred times.
3. We infer factor scores on the fixed held out test set for each of the 100 learned models.
4. We consider the factor scores inferred from each pair of models (*i*, *j*):
  - We use the Hungarian Algorithm [42] to infer the best alignment of factor scores, as measured by correlations.
  - We compute and report the mean correlation between the aligned factor scores.
5. We do this for each model pair and compute the mean of the scores observed.

## Results and discussion

### Generalizability

Tables 1 and 2 show the predictive validity of the Big5 factors, or BLTs (FA5), and models that include age and gender, reported as the mean Pearson r correlation coefficient over 10 random train-test splits for each outcome. For questionnaire-based outcomes, the Big5 factors better predict SWL and depression than our factors. This is driven by strong correlations of SWL and depression with extraversion and neuroticism. The Big5 also outpredicts our factors for the size of the friend network, which again is driven by strong correlations with extraversion. Notably, correlations are equivalent for the Big5 and FA5 in predicting the additional personality questions (Big5Questions). In contrast, as highlighted in the table, our factors better predict income, IQ, and the user likes. For example, on the task of predicting Likes, FA with 5 factors outperforms the baseline by 7 percentage points (60.11–52.6). It is worth emphasizing here that the task of predicting Likes is a challenging task—it essentially involves 20 different classification tasks. Consequently, an improvement of 7 percentage points on this task is promising. Also note that adding age and gender as covariates improves predictive performance (compare FA5+Demog with FA5).

**Table 1:** Predictive performance for behavioral/economic outcomes.

Method	FriendSize	Income	IQ	Likes
Demog	0.052	0.283	0.162	55.50
Big5	0.183	0.037	0.179	52.60
Big5+Demog	0.192	0.278	0.269	56.90
FA5	0.125	0.362	0.361	60.11
FA5 + Demog	0.148	0.375	0.423	61.86

**Behavioral/Economic Outcomes**: Predictive performance for the behavioral, and economic outcomes for the Big5 factors, our learned language based factors (FA5), and demographics (age and gender; DEMOG). We show mean Pearson’s R over 10 random train-test splits for FriendSize, Income and IQ while for Likes we show the mean area under the curve (AUC) over all 20 categories. Language based factors (FA) perform competitively and even outperform questionnaire based factors as highlighted in color.

**Table 2:** Predictive performance for questionnaire based outcomes.

Method	Big5Questions	Sat. W/ Life	Depression
Demog	0.072	0.053	0.103
Big5	0.178	0.486	0.407
Big5+Demog	0.191	0.524	0.424
FA5	0.178	0.165	0.293
FA5 + Demog	0.186	0.207	0.227

**Questionnaire based outcomes** Predictive performance for the questionnaire outcomes for the Big5 factors, our learned language based factors (FA5), and demographics (age and gender; DEMOG). We show mean Pearson R over 10 random train-test splits. Language based factors (FA) do not outperform questionnaire based factors.

Tables 3 and 4 show the best (top) and worst (bottom) Big5 items and LIKES, ranked according to the predictive performance by our BLTs. For Big5 questions, BLTs best predicted openness items, and less predictive of extraversion and conscientiousness items. The strongest items focus on language (e.g., *“have a rich vocabulary”* (see Table 3), suggesting that the BLTs are adequately capturing everyday behavior. For LIKES, BLTs predict general categories (e.g., music genres, children focused), and is least predictive of generic LIKEs which almost all users might like (for example: Youtube, Facebook).

**Table 3:** Questions with the best and worst predictive performance.

**A. 5 questions that the BLTs best predict**.		
**QNO**	Question	R
54	*Am not interested in theoretical discussions* (O-)	0.230
71	*Have a rich vocabulary* (O+)	0.224
64	*Have difficulty understanding abstract ideas* (O-)	0.222
51	*Tend to vote for liberal political candidates* (O+)	0.220
90	*Am filled with doubts* (N+)	0.215
**B. 5 questions that the BLTs least predict**.		
28	*Waste my time* (C-)	0.094
63	*Am the life of a party* (E+)	0.131
43	*Talk to a lot of different people at parties* (E+)	0.133
29	*Dont talk a lot* (E-)	0.135
88	*Find it difficult to get down to work* (C-)	0.139

The Big5 questions on which the BLT factors do the best (top) and worst (bottom) at predicting.

**Table 4:** Likes with the best and worst predictive performance.

**A. 5 Likes that the BLTs best predict**.		
**LIKENO**	Like	AUC
8	*Lady Antebellum, Tim McGraw, NCIS, Kenny Chesney, Country music, Jason Aldean, Walmart, Carrie Underwood, George Strait, Family Feud*	71.04
12	*Glowsticks, Finding Nemo, Being Hyper!, DORY*	68.50
11	*Lil Wayne, Drake, Eminem,T.I., Nicki Minaj, Jersey Shore,Trey*	68.50
10	*I redo high fives if they weren’t good enough the first time, Why do we have to be quiet during a fire drill? Will the fire hear us?*	68.05
14	*The Beatles, Pink Floyd, The Doors, Radiohead, Queen, Nirvana*	65.85
**B. 5 Likes that the BLTs least predict**.		
3	*I hate when im yelling at someone and i mess up what im saying, I would take a bullet for u. Not the head but like in the leg or something*	50.73
0	*YouTube, Facebook, Oreo, Skittles, Coca-Cola, Adam Sandler, Starburst, Starbucks, Music, Toy Story*	51.46
16	*After an arguement I think about clever things I should have said, Your in a good mood, one little thing happens and BAM…. Bad mood*.	54.23
4	*I love days in class when all we do is chill and talk the whole time Get real. No one’s going to form a single line if the building’s on FIRE*.	54.49
1	*When I was little I liked building forts out of pillows and blankets, I like when my scissors glide through the paper so I don’t have to cut*.	54.78

The Categories of Likes that the BLT factors do the best (top) and worst (bottom) at predicting. We show the top LIKES from the clusters for interpretation. AUC = area under the curve

We conclude by emphasizing that the traits learned using FA are *not apriori tuned* to any particular predictive task, and yet perform competitively with traits derived from questionnaires in predicting a variety of outcomes and even outperform questionnaire based traits on behavioral outcomes like Income and IQ, thus underscoring the generalizability of these traits.

### Stability

**Fig 6:**
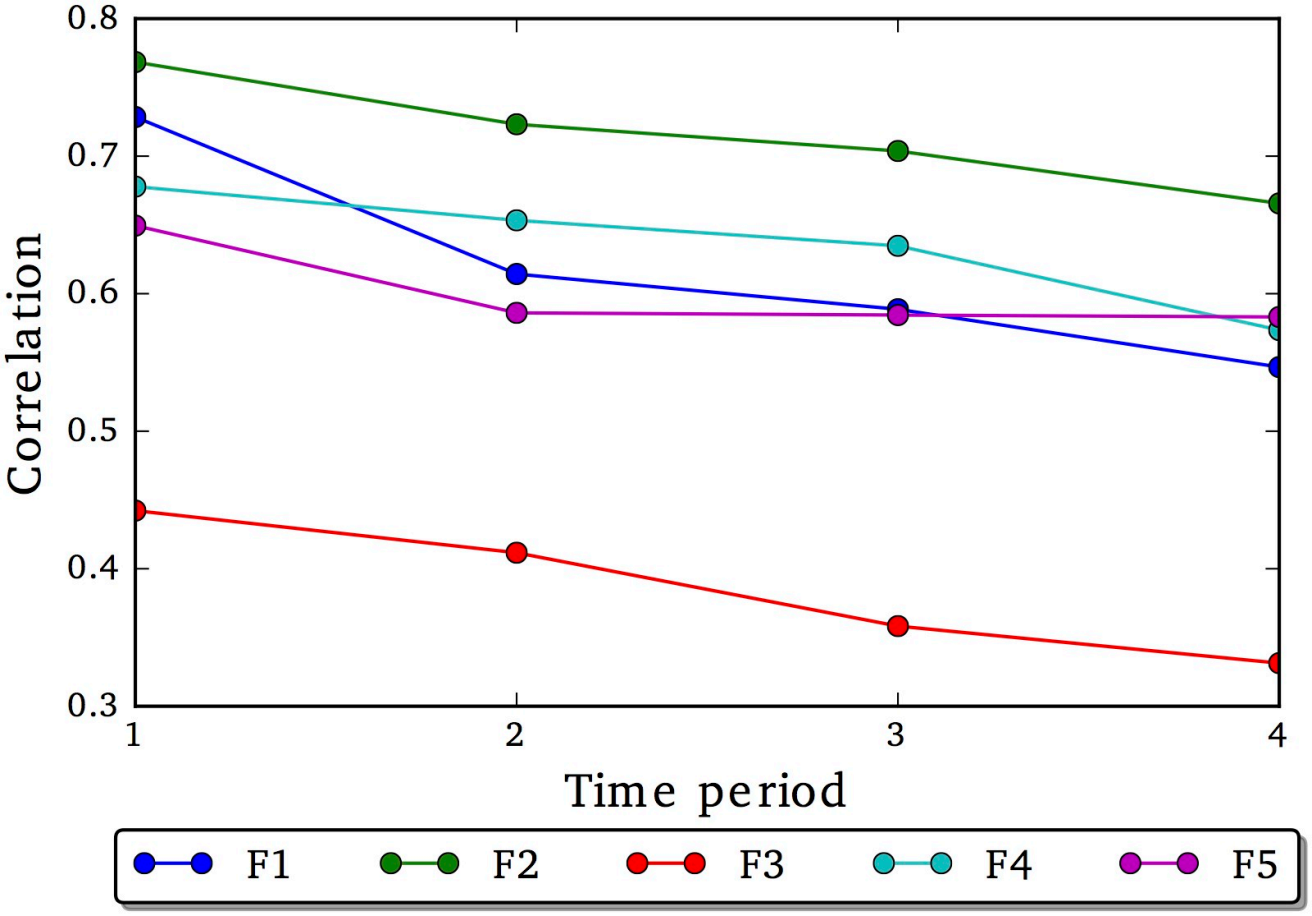
Test re-test validity of our learned factors.

Fig 6 shows the factor correlations at subsequent time periods with the factor scores at the initial point (*t* = 0) over a common set of users. Four of the five factors demonstrate strong correlations, with some decline over subsequent periods, but generally are fairly stable. The exception is F3. The words in this factor (see Fig 1) reflect Facebook behavior (e.g., paste, your status, repost) versus offline behavior (e.g., tomorrow, tonight, sleep, excited). In this case, we believe this factor is capturing less of a trait and more of a temporary “new Facebook user” state.

Finally, for the dropout analysis we computed the mean correlation among factors obtained over multiple runs where a random sample of 20% were dropped before learning the factors. We used the Hungarian algorithm to infer the mapping between factors across multiple runs. There was a very strong average correlation (0.94) among corresponding factors across multiple runs.

Both the test-retest analysis and dropout analysis supports the endurance of the factors across time and sub-populations.

## Limitations

In this first exploration of deriving traits from human behavior manifest on social media, we left many questions unanswered. Our latent traits capture human behavior as revealed in the context of Facebook over 49, 139 US users which may not be generalizable across the globe. We leave it to future work to explore whether similar traits are observed in other contexts. Furthermore, while traditional methods suffer from response biases, our methods have others—for example, although Facebook is used by a majority of Americans there is still no doubt some selection biases in who is on it and people may be projecting how they want to be seen. We see such an approach as a supplement to traditional techniques. Finally, we did not directly address interpretability of our learned factors and did not explicitly impose stability constraints (and revealed we had one more dynamic state-like factor). On the other hand, our goal has been to determine the feasibility of deriving traits from social media behavior, rather than to propose a specific set of traits, akin to the big five, to be used across all contexts. We leave it to future work to rigorously analyze and address these aspects.

## Conclusion

We proposed a method based on **factor analysis** to infer latent personality traits from the everyday language of users on social media. A person is “an organized, dynamic, agentic system functioning in the social world”, with characteristics, behaviors, feelings, and thoughts that are both consistent and variable across time and situations [43]. Individual differences have often been based on questionnaire items, which may not capture the richness of everyday human behavior.

Our method infers latent factors from linguistic behavior in social media—a medium that allows access to large sample sizes; unprompted access to user’s thoughts, emotions, and language; and data-driven approaches. We derived five factors from the language, and demonstrated that these factors are *generalizable* with good predictive power, and *stable* across time and sub-populations. We see this as a stepping stone to deriving a supplement to the popular Big Five personality traits based on large-scale behavioral data rather than questionnaire self-reports. We have demonstrated it is feasible to produce factors from social media language that have predictive and face validity, stability and sometimes generalize better than the Big Five.

## Supporting information

S1 Table Predictive performance on social media tasks for factors without residualization of age and gender for 10 and 30 factors. For comparison, with the questionnaire items, we calculate the 10 aspect scores and 30 facet based scores, using the relevant IPIP items. Demog indicates that age and gender were also added as co-variates to learn predictive models. We show mean Pearson’s R over 10 random train-test splits for FriendSize, Income and IQ while for Likes we show the mean area under the curve (AUC) over all 20 categories. Language based factors (FA) perform competitively and even outperform questionnaire based factors as highlighted in color.
(JPG)

S2 Table Predictive performance on questionnaire based tasks for factors without residualization of age and gender for 10 and 30 factors. For comparison, with the questionnaire items, we calculate the 10 aspect scores and 30 facet based scores, using the relevant IPIP items. Demog indicates that age and gender were also added as co-variates to learn predictive models. We show mean Pearson R over 10 random train-test splits. Language based factors (FA) do not outperform questionnaire based factors.
(JPG)

S3 Table Predictive performance on social media based tasks for factors with residualization of age and gender. We show mean Pearson’s R over 10 random train-test splits for FriendSize, Income and IQ while for Likes we show the mean area under the curve (AUC) over all 20 categories. Language based factors (FA) perform competitively and even outperform questionnaire based factors as highlighted in color.
(JPG)

S4 Table Predictive performance on questionnaire based tasks for factors with residualization of age and gender. Demog indicates that age and gender were also added as co-variates to learn predictive models. We show mean Pearsons R over 10 random train-test splits. Language based factors (FA) do not outperform questionnaire based factors.
(JPG)

S5 Table Predictive performance on social media tasks for factors with residualization of age and gender for 10 and 30 factors. Demog indicates that age and gender were also added as co-variates to learn predictive models. We show mean Pearson’s R over 10 random train-test splits for FriendSize, Income and IQ while for Likes we show the mean area under the curve (AUC) over all 20 categories. Language based factors (FA) perform competitively and even outperform questionnaire based factors (Big5) as highlighted in color.
(JPG)

S6 Table Predictive performance on questionnaire based tasks for factors with residualization of age and gender for 10 and 30 factors. Demog indicates that age and gender were also added as co-variates to learn predictive models. We show mean Pearsons R over 10 random train-test splits. Language based factors (FA) perform do not outperform questionnaire based factors.
(JPG)

S7 Table Predictive performance as a function of vocabulary size. We show mean Pearson’s R over 10 random train-test splits for FriendSize, and IQ while for Likes we show the mean area under the curve (AUC) over all 20 categories. In particular, we learn factors by restricting the vocabulary size to the top *K* words and evaluate these learned factors on their effectiveness on few predictive tasks. In general, we note that predictive performance generally increases with the vocabulary size where we require the vocabulary size to be in the order of a few thousand words to achieve reasonable performance.
(JPG)

S1 Fig Word clouds showing the most/least correlated words for each FA factor as obtained using differential language analysis with age and gender residualized. Residualizing out demographics like age and gender appears to reveal other dimensions of variance like (geography, ethnicity) as illustrated by F5 that reveals a factor highlighting language use of Indians in India with words like india, world-cup.
(TIF)
