# Supplementary figures and images for: Latent human traits in the language of social media: An open-vocabulary approach
Source: PLoS One. 2018 Nov 28;13(11):e0201703. doi: 10.1371/journal.pone.0201703 (PMC6261386; doi:10.1371/journal.pone.0201703)

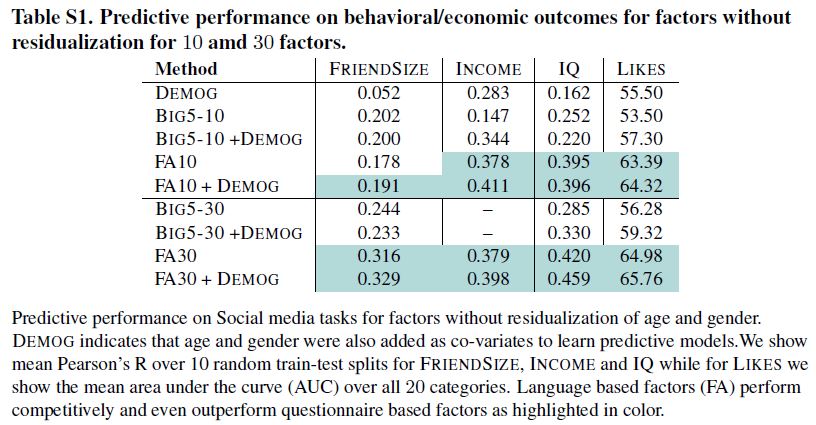

Supplement: S1 Table — For comparison, with the questionnaire items, we calculate the 10 aspect scores and 30 facet based scores, using the relevant IPIP items. Demog indicates that age and gender were also added as co-variates to learn predictive models. We show mean Pearson’s R over 10 random train-test splits for FriendSize, Income and IQ while for Likes we show the mean area under the curve (AUC) over all 20 categories. Language based factors (FA) perform competitively and even outperform questionnaire based factors as highlighted in color. (JPG) [file pone.0201703.s001.JPG]

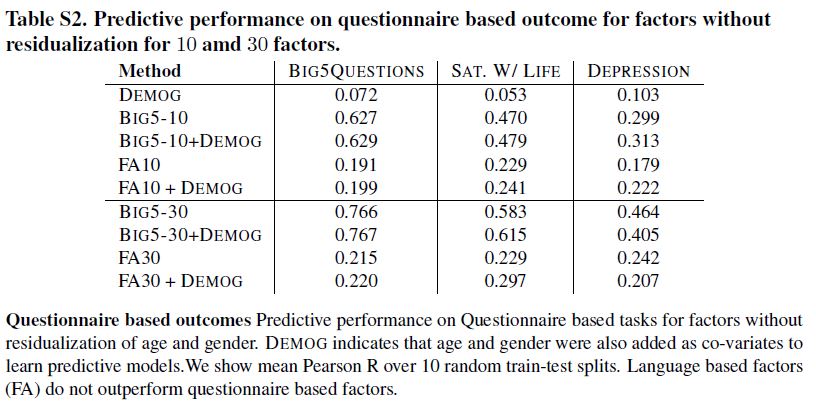

Supplement: S2 Table — For comparison, with the questionnaire items, we calculate the 10 aspect scores and 30 facet based scores, using the relevant IPIP items. Demog indicates that age and gender were also added as co-variates to learn predictive models. We show mean Pearson R over 10 random train-test splits. Language based factors (FA) do not outperform questionnaire based factors. (JPG) [file pone.0201703.s002.JPG]

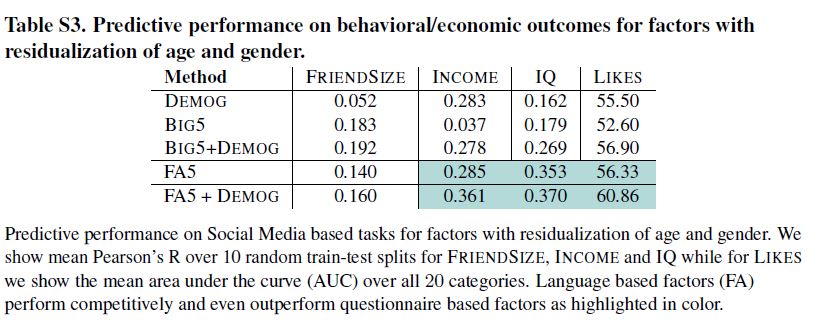

Supplement: S3 Table — We show mean Pearson’s R over 10 random train-test splits for FriendSize, Income and IQ while for Likes we show the mean area under the curve (AUC) over all 20 categories. Language based factors (FA) perform competitively and even outperform questionnaire based factors as highlighted in color. (JPG) [file pone.0201703.s003.JPG]

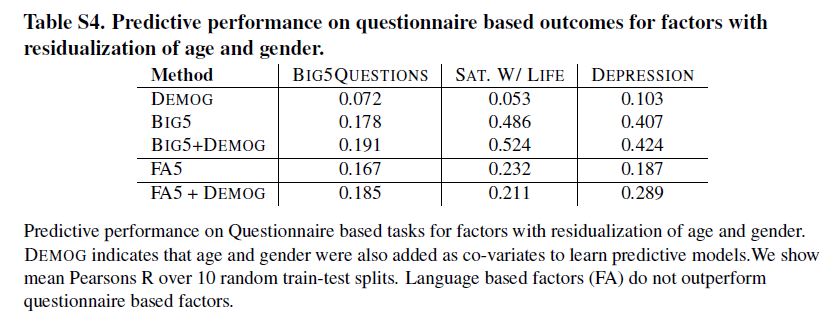

Supplement: S4 Table — Demog indicates that age and gender were also added as co-variates to learn predictive models. We show mean Pearsons R over 10 random train-test splits. Language based factors (FA) do not outperform questionnaire based factors. (JPG) [file pone.0201703.s004.JPG]

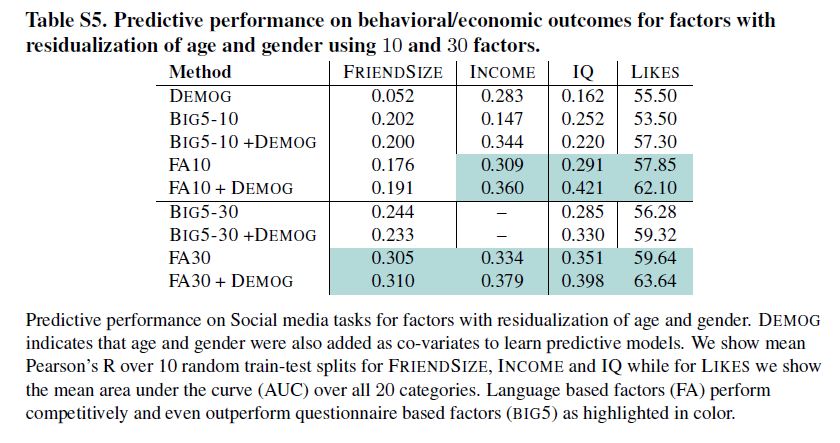

Supplement: S5 Table — Demog indicates that age and gender were also added as co-variates to learn predictive models. We show mean Pearson’s R over 10 random train-test splits for FriendSize, Income and IQ while for Likes we show the mean area under the curve (AUC) over all 20 categories. Language based factors (FA) perform competitively and even outperform questionnaire based factors (Big5) as highlighted in color. (JPG) [file pone.0201703.s005.JPG]

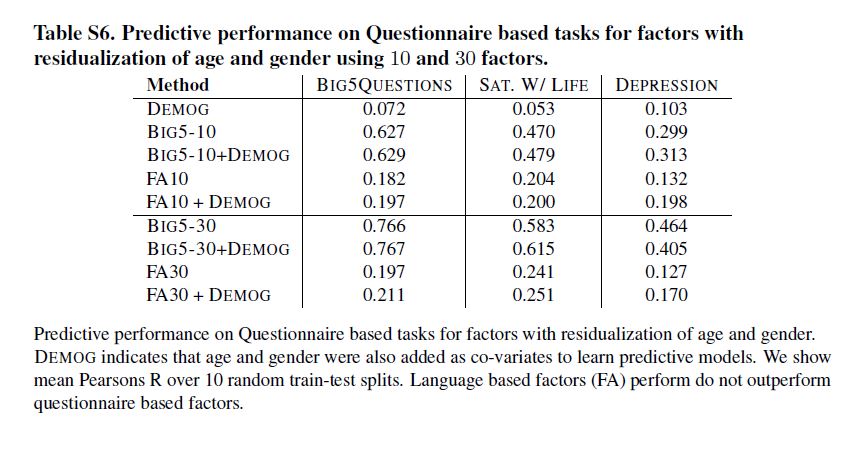

Supplement: S6 Table — Demog indicates that age and gender were also added as co-variates to learn predictive models. We show mean Pearsons R over 10 random train-test splits. Language based factors (FA) perform do not outperform questionnaire based factors. (JPG) [file pone.0201703.s006.JPG]

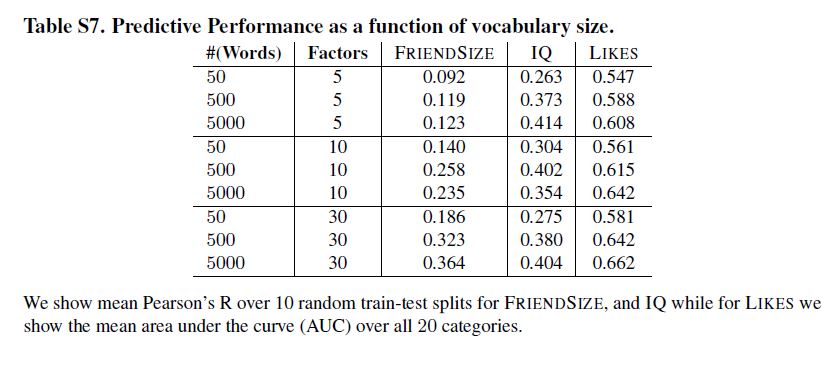

Supplement: S7 Table — We show mean Pearson’s R over 10 random train-test splits for FriendSize, and IQ while for Likes we show the mean area under the curve (AUC) over all 20 categories. In particular, we learn factors by restricting the vocabulary size to the top K words and evaluate these learned factors on their effectiveness on few predictive tasks. In general, we note that predictive performance generally increases with the vocabulary size where we require the vocabulary size to be in the order of a few thousand words to achieve reasonable performance. (JPG) [file pone.0201703.s007.JPG]

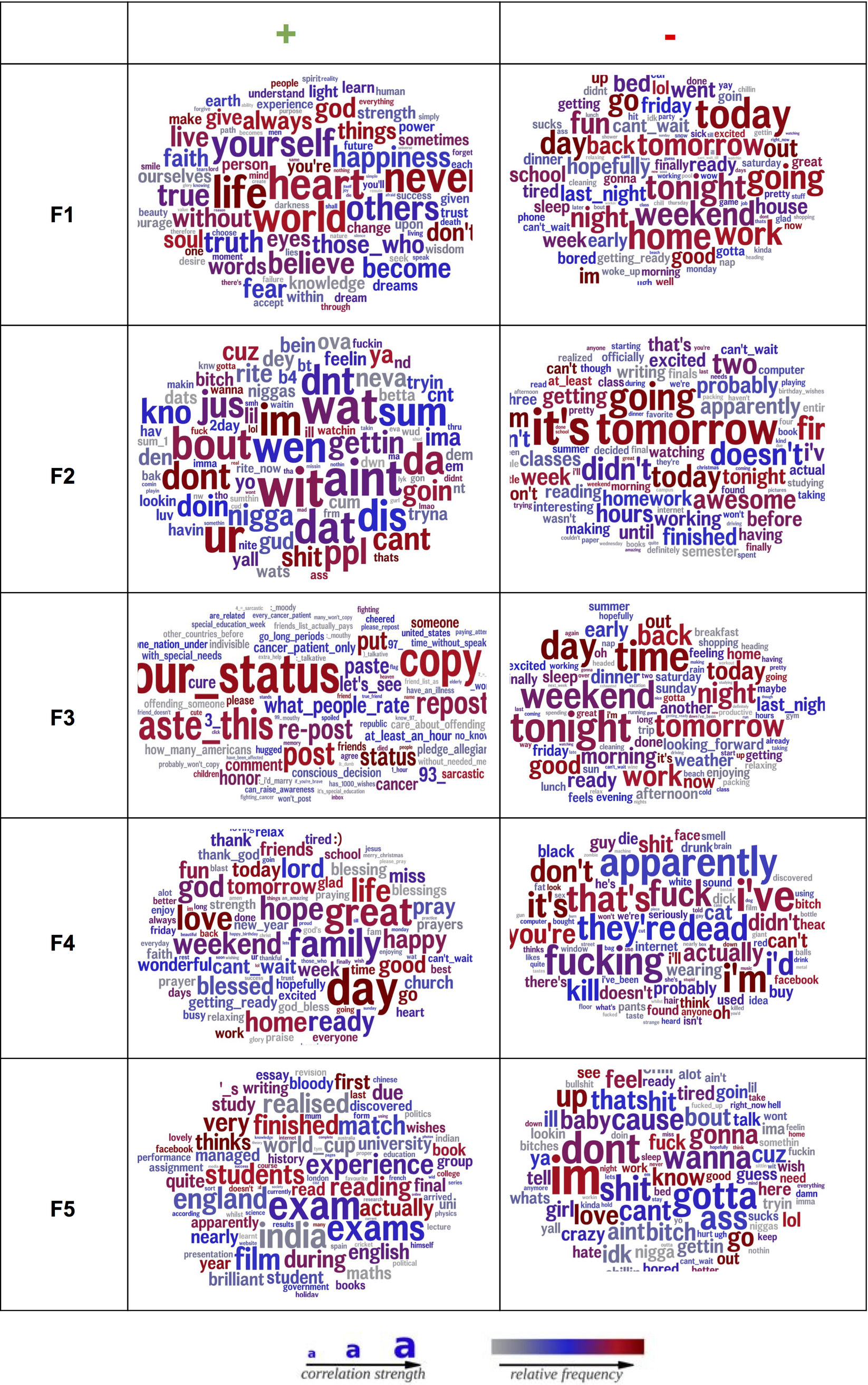

Supplement: S1 Fig — Residualizing out demographics like age and gender appears to reveal other dimensions of variance like (geography, ethnicity) as illustrated by F5 that reveals a factor highlighting language use of Indians in India with words like india, world-cup. (TIF) [file pone.0201703.s008.tif]
